# Emergence and Dissemination of the Avian Infectious Bronchitis Virus Lineages in Poultry Farms in South America

**DOI:** 10.3390/vetsci12050435

**Published:** 2025-05-02

**Authors:** Vagner Ricardo Lunge, Diéssy Kipper, André Felipe Streck, André Salvador Kazantzi Fonseca, Nilo Ikuta

**Affiliations:** 1 Simbios Biotecnologia, Cachoeirinha 94940-030, RS, Brazil; diessykipper@hotmail.com (D.K.); fonseca@simbios.com.br (A.S.K.F.); ikuta@simbios.com.br (N.I.); 2 Institute of Biotechnology, University of Caxias do Sul (UCS), Caxias do Sul 95070-560, RS, Brazil; afstreck@ucs.br

**Keywords:** IBV, phylogeny, genetic type I (GI), surveillance, molecular epidemiology

## Abstract

Infectious bronchitis virus (IBV) is a chicken pathogen present in commercial poultry farms worldwide. It is classified within the species *Avian coronavirus*, genus *Gammacoronavirus*. As with other members of the family *Coronaviridae*, it has a single positive-sense RNA genome with 27.6 Kb and presents viral particles with a typical crown-like aspect due to the spike (*S*) transmembrane glycoprotein. IBV has a remarkable capacity for genetic recombination and mutation, resulting in many genotypes and antigenic variants over evolutionary time. Currently, it is classified into nine genetic types (GI to GIX) and 41 (1 to 41) lineages disseminated worldwide. In South America, IBV was first identified in early commercial poultry production ventures in Brazil in the 1950s. Since then, this virus has been frequently detected in commercial South American poultry farms, being classified into serotypes in the first decades and genotypes more recently. IBVs of the Massachusetts (Mass) serotype were initially detected and vaccine strains of this serotype were used extensively on commercial poultry farms. Other serotypes/genotypes were identified later, with almost all of them classified in the current genetic type I (GI). In addition, five GI lineages (GI-1, -11, -13, -16, and -23) have been associated with the main infectious bronchitis outbreaks in the continent, with some variations in the occurrence according to the countries and the period of time. Molecular epidemiological surveillance of IBV genetic types and lineages is necessary to anticipate potential outbreaks, revealing patterns of viral evolution and dissemination, as well as to guide the selection of appropriate vaccine strains and immunization programs.

## Simple Summary

Avian infectious bronchitis virus (IBV) is a pathogen that affects chickens, causing respiratory disease and other clinical manifestations. It is a coronavirus with several genetic and antigenic variants that display different levels of pathogenicity and have been detected and characterized in commercial poultry farms worldwide since the 1930s. Infectious bronchitis outbreaks have been controlled through the use of vaccines (live and inactivated), with different strains, over time. Like other coronaviruses, different IBV variants (genetic types/lineages) have emerged and increased/decreased in frequency with international trade and poultry practices, including vaccination. This review reports the genetic and antigenic diversity of IBV and the methods used to classify and identify it into genetic types and lineages. In addition, the emergence, dissemination, and current epidemiological situation of the main five lineages of this virus in South American poultry farms are described. These molecular epidemiological surveillance data are necessary to identify IBV circulating lineages and implement efficient biosafety measures and vaccination programs to control this concerning chicken pathogen.

## 1. Introduction

Avian infectious bronchitis virus (IBV) is a pathogen of chicken (*Gallus gallus*) that causes severe respiratory, renal, and reproductive disorders in commercial poultry flocks worldwide [1]. IBV was the first member of the *Coronaviridae* family to be isolated and propagated in the laboratory in the 1930s [2]. It was soon associated with outbreaks of severe respiratory disease with high mortality (hence the name infectious bronchitis) that occurred in poultry flocks from farms located in several states (Massachusetts, Connecticut, Arkansas, and others) in the United States (US). IBV was also detected in symptomatic poultry flocks in Europe, Asia, America, Australia, and Africa, not long after [3,4]. The high economic impact of IBV on poultry production has forced the implementation of control procedures, such as biosecurity measures and vaccination, to prevent the entry of pathogenic viral strains into many commercial farms [5]. The first vaccines were live or inactivated viruses, which were isolated from clinical samples, propagated in the laboratory, and attenuated by multiple passages in embryonated chicken eggs (ECE) [2]. They were produced and used in different US states in the 1950s and some were distributed globally, highlighting the Massachusetts (Mass) vaccine strains [6].

The occurrence of IBV is now well established in commercial poultry farms as well as backyard chickens worldwide, with several genetic types, lineages, and variants according to geographic regions, poultry/bird managements, and period of time [3,5,6]. More and less virulent IBVs affect both broilers and laying hens, causing significant economic losses to local or global poultry production systems. Due to its socioeconomic impact and the damage to bird health associated with mortality and reduced egg production, infectious bronchitis is included in the WOAH (World Organization for Animal Health) list of notifiable diseases [7]. Significant and measurable impacts on the broiler and breeder industries have been reported, with an age-dependent increasing trend and an association with multiple circulating genetic types and lineages [8]. As a consequence, different IBV vaccines have been produced, tailored to specific strains worldwide, including three main types: live attenuated, inactivated (killed), and recombinant vaccines [9].

In South America, this disease has also caused outbreaks in poultry farms for decades. Due to the different geography, climate, and animal health policies in different countries, poultry farming processes and disease control have specificities in each geographic region. The different evolutionary poultry farming histories, as well as the limited cross-protection of the used vaccines in the countries, have also contributed to the emergence of different IBV field variants over time [10]. As a result, IBV spreading waves occurred in different ways in most poultry-producing regions of this continent [11,12,13,14]. Despite the widespread and increasing use of vaccines and other control procedures, field variants are still very prevalent in commercial poultry flocks. Furthermore, the international trade in animals, biologicals, and other products in broiler and layer production chains has enabled the dissemination of other IBV genetic types and lineages more recently [3,4].

This review aims to describe some important characteristics of IBV, explain the reasons for the genetic and antigenic diversity, describe the methods to classify and identify IBVs, and report the emergence and dissemination of the main genetic types/lineages in poultry farms in South America. The current situation of IBV diversity in the main poultry-producing regions of this continent is also highlighted in the last sections.

## 2. Taxonomy and Molecular Structure

**Figure 1:**
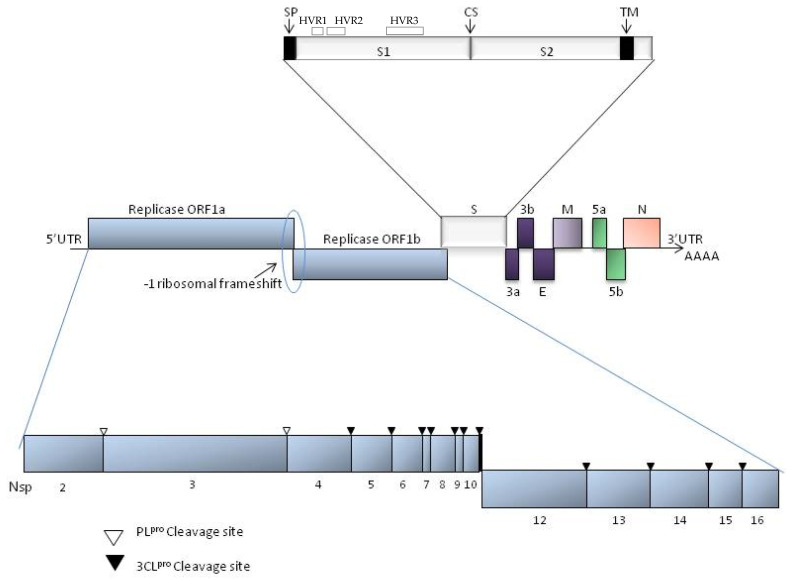
Classical genome organization of IBV. The genome is 27,620 nt long, excluding poly (A) tract. Middle: ten genes and ORFs (open reading frames). Ribosomal frameshift is indicated. Top: putative domains of ORF1a/1b polyprotein: nsp-non-structural protein. Bottom: details of spike protein; SP-signal peptide, CS-spike protein cleavage, TM-transmembrane domain of spike protein. In the spike protein subunit 1 (S1) the three hypervariable regions (HVR1, HVR2, and HVR3) are indicated.

IBV is currently classified within the species *Avian coronavirus*, subgenus *Igacovirus*, genus *Gammacoronavirus*, subfamily *Othocoronavirinae*, family *Coronaviridae*, order *Nidovirales* [15]. The IBV genome is a single positive-sense RNA strand about 27.6 Kb in length, genetically organized as follows: 5′UTR-1a/1ab-S-3a-3b-E-M-5a-5b-N-3′UTR (Figure 1).

As a positive-sense RNA genome, it has 5′ and 3′ untranslated regions (5′ and 3′-UTRs) that simulate 5′-cap and a 3′-poly(A) tail from mRNAs, acting in the translation of the replicase polyproteins [10]. These two regions also interact with viral replicases and potentially other proteins in the host ribosomes [16]. The remaining genome encodes more than 20 non-structural and structural proteins/enzymes synthesized with the translation of the RNA genome. Around 15 non-structural proteins (2 to 16) are encoded by the two open reading frames (ORFs) ORF1a (~3934 aa) and ORF1b (~2653 aa) [10]. All these proteins are involved in the viral replication process in the host cell. In addition, four main structural proteins compose the virion particles: *S* (spike; ~1163 aa), *E* (envelope; ~110 aa), *M* (membrane; ~226 aa), and *N* (nucleocapsid; ~410 aa). Finally, accessory proteins 3a, 3b, 5a and 5b (~58 aa; ~65aa; ~66aa; ~83aa, respectively) are also produced. They are not directly required for replication or viral structure and are supposed to act by immune modulation of the infection [11].

**Figure 2:**
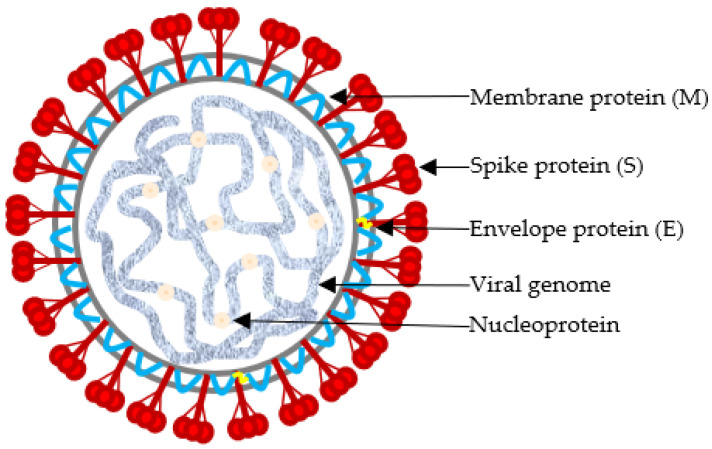
Schematic diagram of the structure of the infectious bronchitis virus showing envelope protein, spike protein, membrane protein, vial genome, and nucleoprotein.

In the structure of the viral particles, *S* is a transmembrane glycoprotein organized in trimers, which determines the typical crown-like aspect of coronaviruses, observable at electron microscopy [17]. It is post-translationally cleaved into two subunits, *S1* and *S2*, with approximately 535 and 625 amino acids, respectively. The *S1* glycoprotein is necessary for adsorption to the cellular receptor and *S2* is critical for virus entry into the host cell. The other surface proteins *M*, *N*, and *E* are involved in virus morphogenesis and assembly [10] (Figure 2).

## 3. Genetic and Antigenic Diversity

IBV and all members of the family *Coronaviridae* have a remarkable capacity for genetic recombination and mutation compared to other RNA viruses. IBV genome changes due to two main biological processes: genetic shift and genetic drift. Genetic shift occurs when there is a wider genetic recombination event (involving large parts of the genomes or genes) between two different IBV strains, also promoting drastic changes in the viral structure and the sudden emergence of new variants with significant modifications, including antigenic and virulence aspects. Genetic drift occurs due to more specific nucleotide mutations (most of them in a single nucleotide) that usually result in subtle variations in the genome, but which, when added together over time, also affect antigenicity and virulence. The natural occurrence of these two main genetic processes, often acting together, is what has enabled the emergence of evolutionarily successful IBV variants over time [5].

The recombination capacity is due to a copy-choice mechanism, where the RNA-dependent RNA polymerase switches between RNA templates (whenever the cell is infected with two different coronaviruses) during synthesis, producing chimeric RNA with mixed ancestry [18]. Some regions of all coronavirus genomes appear to be more susceptible to recombination than others, forming recombination “hot spots” [5]. In the IBV genome, the more studied “hot spots” are located in the *S* gene and they are known as hypervariable regions 1 (HVR1), 2 (HVR2), and 3 (HVR3). Considering the whole peptide chain of the Spike protein, they are located from amino acids 38 to 67, from amino acids 91 to 141, and from amino acids 274 to 387, respectively (Figure 1).

The *S* gene, especially the *S1*, is primarily responsible for the antigenicity of the virus, being the main target of the host immune response [19]. Recombination in the *S* gene can lead to the emergence of new genetic variants, as well as new taxa capable of infecting and causing disease in different host species [5,6]. In addition to the *S* region, recombination also occurs in regions of the IBV genome, such as Nsp2, Nsp3, and Nsp8 in the ORF1a, as well as Nsp12 and Nsp16 in the ORF1b. This recombination plays a crucial role in viral evolution and may impact replication, immune escape, and adaptation of IBV to new hosts [5,6,20].

Furthermore, mutations are fundamental in IBV evolution, especially point mutation. IBV has an estimated substitution rate of 10^−4^–10^−5^ substitutions/site/year, providing a remarkable evolutionary potential [21,22,23]. In the IBV genome, a high error rate and limited proofreading contribute to nucleotide mutations and consequently amino acid substitutions [5].

IBV genetic diversity is directly related to its antigenic variability, mainly due to alterations in the *S* gene. In particular, the *S1* subunit is the more variable region in the protein, so the *S1* gene is commonly sequenced in molecular assays to type IBV strains [6]. The emergence of new IBV lineages/serotypes is largely due to the instantaneous recombination events and accumulation of mutations in the *S* gene over time, resulting in structural modifications in the *S* protein. These variations alter the antigenic determinants of IBV, making disease control an ongoing challenge in poultry farming [5,10].

## 4. Classification and Identification

IBVs have been classified since their first identification in the US. At that time, methods for the characterization of IBVs were based on classic virological techniques, including viral isolation in chicken embryos and subsequent observation of characteristic lesions in the allantoic cavity. In addition, hemagglutination inhibition (HI) and serum neutralization (SN) tests should be used to determine the antigenic relationship between different strains [10].

These first serological methods evaluated mainly the *S* protein, the target of neutralizing antibodies. These tests, such as HI and VN (viral neutralization), allow IBVs in US poultry farms to be grouped into serotypes according to the state of the detection, highlighting Massachusetts (Mass), Arkansas (Ark), and Connecticut (Conn), among others. Serological classification was essential to choose some vaccine strains and to study cross-protection between them and field variants [9]. By comparing antibody titers against different strains, it was possible to estimate the degree of protection conferred by a vaccine variant against a strain circulating in the field. In addition, serological tests have been valuable tools for monitoring the immune response of the poultry population over time, aiding in decisions for disease control [4,10,24].

Developments in molecular biology and the introduction of the polymerase chain reaction (PCR) and DNA sequencing methods allowed the rapid detection and differentiation of IBVs, including vaccine strains or field variants. First, molecular methods were designed to evaluate small regions in the *S* gene, highlighting HVRs due to more informative, diverse sequence data. Currently, more powerful complete *S1* gene and IBV whole-genome analyses have been used to classify and identify genetic types, lineages, and strains. This progress in molecular biology methods in recent decades has contributed to a more complete scenario, enabling the classification of IBV in different genetic types and dozens of lineages and tracking the emergence and dissemination of novel field variants.

The current IBV classification is based on complete *S1* gene sequencing. IBV is classified into nine genetic types (GI to GIX) and at least 41 (1 to 41) lineages defined through comprehensive studies that analyzed samples from different parts of the world. The nine genetic types represent significant variations in the viral genome sequence, reflecting evolution and adaptation to different hosts and geographic environments. In addition, lineages are subdivisions within these genetic types, reflecting more specific variations that have arisen over time due to mutation and genetic recombination [6,25,26]. IBV GI is most associated with outbreaks of infectious bronchitis on commercial poultry farms and is present in virtually all commercial poultry production sites worldwide, with the following lineages showing significant presence by geographic region: GI-1, -12, -13, -14, -16, -19, -21, -23, and -26 in Africa; GI-1, -2, -3, -4, -7, -13, -15, -18, -19, -22, -24, -28, and -29 in Asia; GI-1, -12, -13, -16, -19, and -23 in the Middle East; GI-1, -12, -13, -14, -16, -19, -21, and -23 in Europe; GI-1, -2, -3, -8, -9, -17, -25, and -27 in North America; GI-1, -5, -6, and -10 in Oceania; and GI-1, -9, -11, -13, -16, -17, -23, and -30 in South America [25,27].

## 5. IBV Emergence and Dissemination in South America

IBV was first identified in South America in the early commercial poultry production ventures in Brazil in the 1950s [28]. IBV antibodies against the Massachusetts serotype were detected in chickens using the SN test. Tissue samples from these chickens were also inoculated into ECE, which showed typical signs of IBV infection. These early cases were then identified as Mass-type viruses [29].

After its emergence in Brazil, IBV was not immediately associated with severe respiratory disease with high mortality in poultry flocks. At that time, chicken production was not intensive, being based on “backyard” farming [29]. However, Brazilian poultry production increased significantly afterward, mainly in the states of São Paulo and Santa Catarina. The focus was on supplying chicken and eggs to domestic and international markets, with exports to countries in the Middle East. Poultry farms initially produced almost exclusively older chickens. With a more pronounced intensification of chicken production in the 1970s, severe IBV outbreaks were observed in laying hens (with reductions in the production and quality of eggs) and broiler hens (respiratory problems and lesions in the respiratory and renal systems) [29].

In these same decades, IBV outbreaks were also reported in other South American countries with growing poultry production, such as Colombia, Chile, and Argentina. In Colombia, the first isolation of IBV was reported in 1963 from samples of 25-day-old broilers and 5-month-old layers with respiratory signs [30]. Despite the use of some vaccines (based on D274/D207, Ark, and Conn strains), these outbreaks of infectious bronchitis were not completely controlled [30]. In the 2000s, isolates corresponding to serotypes Conn and Mass, as well as four isolates with unique or indigenous lineages, were additionally identified in this same country [31]. In Chile, IBV has been diagnosed based on clinical, pathological, and serological signs since 1967 [32]. In the 1980s, the virus became a serious problem in commercial flocks, with the detection of the Mass and Conn serotypes and new variants, against which the H120 vaccine showed low protection [33]. In Argentina, IBV has also been endemic for a long time and its control is carried out through vaccination, mainly using vaccines based on the Mass strain [34].

**Figure 3:**
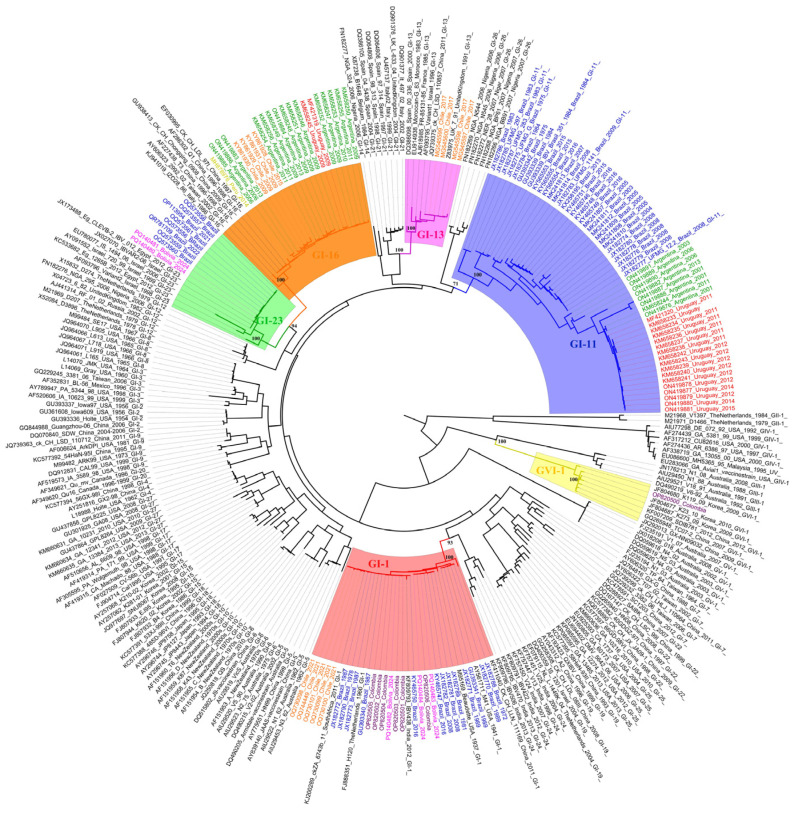
Phylogenetic tree constructed using complete sequences of the IBV S1 region obtained from the NCBI database. The selected representative sequences of groups GI to GVI, according to the Valastro et al. [6] classification, and include all complete IBV S1 sequences available for South American countries. Color labels denote country of origin: green = Argentina, pink = Bolivia, blue = Brazil, orange = Chile, purple = Colombia, yellow = Peru and red = Uruguay. Clade colors denote groups: red = GI-1, blue = GI-11, pink = GI-13, orange GI-16; green = GI-23 and yellow GVI-1.

In summary, South America has a variety of lineages of avian infectious bronchitis virus, some of which are unique to the region [11,14,22,30,33,34]. A phylogenetic analysis using complete sequences of the IBV *S1* region obtained from the NCBI (National Center for Biotechnology Information) database demonstrates different GI lineages in South American countries as well as a novel genetic type (GVI) detected in Colombia (Figure 3). Importantly, IBV GI-1 is largely distributed in almost all countries, while GI-11 is mostly detected in Argentina, Brazil, and Uruguay, GI-13 in Brazil, GI-16 in Argentina, Chile, Peru, and Uruguay, and the most recent GI-23 in Bolivia and Brazil. This result indicates that each GI lineage has its evolutionary history on this continent, as addressed in the next sections.

### 5.1. GI-1

IBV GI-1 was the first lineage identified in South America. It probably remains one of the most widely distributed lineages due to the widespread use of live homologous vaccines derived from Mass strains on this continent for over 50 years [6]. The first vaccine for IBV control was developed in the 1950s, using the van Roeckel M-41 strain, in the US [35]. It served as the parental strain for most Mass-type vaccines used at that time in North America. In Europe, the H strain (also from Mass serotype and GI-1) was used to develop the H52 and H120 vaccines in the Netherlands in the early 1960s [36]. Currently, live attenuated vaccines based on the M41 and H strains are still largely administered in commercial poultry farms worldwide [6,11].

In South America, GI-1 has been reported in poultry farms in Argentina, Brazil, Bolivia, Chile, and Colombia since the 1950s [14,22,30,33,34,37]. In Brazil, IBV GI-1 was first identified in 1957 [28]. With more concerning IBV outbreaks in the 1970s and 1980s, the H120 and H52 strains were imported to produce Mass live vaccines and used to immunize poultry flocks. Subsequently, these H strains have been largely used in commercial poultry farms in Brazil and other South American countries [29,31,34].

However, IBV GI-1 has also been detected in poultry flocks with infectious bronchitis signs in South America since the early 2000s. In Colombia, this lineage was associated with outbreaks of chickens presenting respiratory signs, such as coughing and rales, in addition to a decrease in egg production [31]. In Argentina, GI-1 outbreaks were also reported, with birds presenting intense respiratory symptoms and tracheal lesions [34]. In Brazil, typical clinical cases of infectious bronchitis, such as tracheitis, airsacculitis, and a decrease in zootechnical performance, were observed, with the identification of GI-1 strains [29]. More recently, IBV GI-1 strains associated with outbreaks in Colombian poultry farms were more completely characterized through whole-genome analysis, demonstrating high viral genetic diversity [30]. IBV GI-1 field strains were also detected in broiler flocks with acute respiratory signs in Bolivia [14].

A more detailed evaluation of the IBV GI-1 cluster in the phylogenetic tree has demonstrated some subtle variations in the branches due to slight nucleotide divergences, suggesting vaccine GI-1 modifications in the field (Figure 3). It has already been shown that vaccine usage results in fast evolutionary rates in field viruses. IBV can also be shed for long periods and vaccine viruses persist in the field and revert to cause disease [38].

### 5.2. GI-11

IBV GI-11 lineage seems to be almost exclusive to South America. A previous study has demonstrated that it probably emerged in Brazil in the 1950s [22]. Furthermore, other reports have described its predominance in poultry farms (broilers, layers, breeders) with chickens presenting different clinical signs (respiratory distress, decreased egg production, kidney lesions). IBV outbreaks by GI-11 have been reported in different poultry-producing regions in this country from the 1990s to today [11,22,23,39,40,41]. IBV GI-11 population dynamics seem to remain largely stable over time [22]. In 2016, a GI-11 live-attenuated vaccine strain was launched to control this lineage [22]. Later, field and vaccine strains were detected in chickens in commercial poultry farms [41].

This lineage has been frequently detected in Argentina and Uruguay too [34,40]. IBV outbreaks by GI-11 were reported in Argentina between 2001 and 2008. Chicken presented respiratory distress, decreased egg production, and kidney lesions [34]. The presence of GI-11 was identified in poultry samples in Uruguay in 2019. Clinical signs included bronchitis and low feed conversion [40]. The emergence of GI-11 was more recently reported in commercial broiler flocks in Colombia in 2021, associated with intense respiratory signs and increased mortality [12].

The assertion that GI-11 is exclusive to South America raises important questions about the role of geographic isolation in the emergence and evolution of this lineage. Several factors may have contributed to its regional specificity, including limited poultry trade with other continents, vaccination strategies, and unique ecological and environmental conditions that may have shaped this IBV lineage evolution. In addition, genetic drift and selection pressures from local IBV strains may have facilitated the establishment of GI-11 as a dominant lineage in Brazil and later in other South American countries [25,27]. A recent study demonstrated that GI-11 is not more restricted to this continent, since it was detected in birds from southern Africa between 2010 and 2020 [42]. Additional molecular epidemiological studies are needed to confirm the dissemination of this lineage and to understand the mechanisms involved in its emergence and persistence.

### 5.3. GI-13

IBV GI-13 has been reported in two South American countries, Brazil and Chile, in this century [22,37]. Noteworthy, GI-13 field variants were previously identified as the cause of severe outbreaks in Europe and specific vaccine strains (793B, 4/91 and CR88) were developed to control this lineage in the 1990s and also used in other poultry-producing continents [43,44]. In Brazil, GI-13 was detected in poultry flocks vaccinated with the Mass strain, presenting respiratory signs and a drop in egg production in 2007 and 2008 [45]. After that, there were no more reports of infectious bronchitis caused by GI-13 in commercial poultry farms, suggesting that outbreaks caused by this lineage were rare and self-limiting [22]. In Chile, broilers flocks with GI-13 were identified with clinical signs such as respiratory distress, tracheitis and renal alterations, mainly after the introduction of specific vaccine strains against this lineage [37].

### 5.4. GI-16

IBV GI-16 seems to have emerged probably in Asia in around 1979 [40]. Since then, it has spread to different continents, including South America [11]. It quickly became one of the four most prevalent IBV lineages, alongside GI-1 (Mass), GI-13 (793B), and GI-19 (QX) [6]. The GI-16 lineage comprises two genetic groups with a shared origin [6,46]: strain 624/I was identified in Italy in 1993 [47], while strain Q1 (also known as A/SAII) was discovered in China in 1996 [48].

In South America, IBV GI-16 has been detected in Argentina, Chile, Colombia, Peru, and Uruguay (Figure 3) [11,37,40,49,50]. In Chile, IBV GI-16 poultry outbreaks were associated with respiratory signs, decreased production, and renal impairment in the affected chicken flocks [37]. In Uruguay, birds infected with this lineage had respiratory symptoms and low zootechnical performance [40]. The persistent presence of GI-16 was confirmed in these and other South American countries, with flocks presenting respiratory problems and low uniformity [11]. In 2022, it was reported that new GI-16 lineages emerged through recombination and mutation events, with significant clinical impact in chickens with respiratory compromise and immunosuppression [49]. More recently, GI-16 variants were identified in Peru, being associated with severe respiratory signs. It was also suggested that the local evolution of the lineage was due to specific alterations in the *S1* gene and respective protein [50].

### 5.5. GI-23

IBV GI-23 lineage emerged in the Middle East and has been increasingly detected in severe respiratory infections from broiler flocks worldwide [5]. This lineage reached Europe and Africa in the last few decades and it was first reported in Brazil in the last few years [6,13,41,51]. A temporal study demonstrated that IBV GI-23 was introduced in Brazil and probably in South America between 2017 and 2019 [52].

Clinical cases related to IBV GI-23 have been characterized by severe respiratory clinical signs, renal lesions, and increased mortality [13]. The pathogenicity of one GI-23 strain isolated in Brazil was more deeply evaluated, with infected birds presenting clinical signs such as tracheitis and airsacculitis [51]. In addition, GI-23 seems to have tropism for extrapulmonary tissues, such as the kidneys and reproductive tract, which can result in the reduction of egg production in layers and breeders [13,51].

In 2024, the detection and molecular characterization of GI-23 in broiler chickens in Bolivia was reported, with respiratory clinical symptoms and a drop in zootechnical performance [14]. This lineage has been controlled by specific immunization with the development of homologous vaccine strains [5].

### 5.6. Current Situation

The current situation of the molecular epidemiology of IBV in South America reflects the emergence and dissemination of the five main GI lineages described here at different times in the continent, as well as the implementation of different vaccine strains and immunization schemes in the last decades. GI-1 is widely disseminated, due to the first dissemination of IBV on the continent and the massive use of Mass live vaccine strains. In addition, GI-11 is more frequent in Brazil, while GI-16 predominates in Argentina, Chile, and Colombia, probably due to different evolutionary histories related to independent introductions and adaptations to poultry production chains in these countries. GI-13 has also been detected (but very rarely), while GI-23 has emerged more recently and is spreading rapidly in many countries.

It is also important to highlight the introduction of specific vaccine strains and programs to improve protection against the predominant GI-11 and GI-16 lineages in the last two decades since these lineages have a low antigenic relationship with the Mass strains previously used as vaccines [11]. Different immunization schemes have also been introduced, such as the combined use of the BR1 (GI-11) and Mass (GI-1) strains of IBV to provide high levels of protection against heterologous strains such as 793B, QX, Q1, and Variant 2 [53]. The recent emergence of IBV GI-23 brought an additional challenge and more vaccine strains and immunization programs had to be implemented to control it [5].

**Figure 4:**
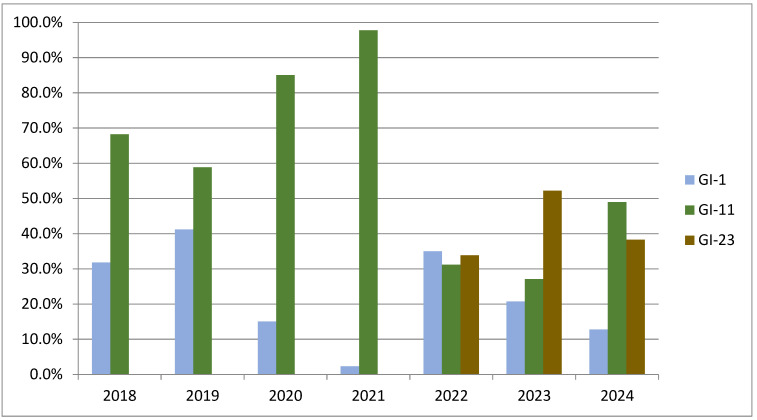
Percentage distribution of IBV genetic groups GI-1, GI-11, and GI-23 over the years (2018–2024) in Brazil. The *Y*-axis represents the percentage of occurrence, while the *X*-axis indicates the years analyzed. The blue bars correspond to the GI-1 group, the green bars to the GI-11 group, and the brown bars to the GI-23 group. There is a variation in the prevalence of the groups over time, with emphasis on the peak of GI-11 in 2021 and the increase in GI-23 in more recent years.

The dynamics of frequency changes of IBV lineages can be observed from detection data of GI-1, GI-11, and GI-23 in Brazil over a 7-year period (2018 to 2024) (Figure 4). The GI-11 lineage (green) appears to predominate throughout the first years of monitoring, reaching a peak in 2021. This lineage shows a decline in percentage frequency in 2022, when the GI-23 lineage (brown) began to emerge, maintaining a considerable frequency until 2024. The GI-1 IBV lineage (blue) showed relative stability, with a slight increase in 2022 and subsequent decline. These data reinforce the dynamic epidemiological pattern of IBV lineages, with changes in predominance over the years, mainly influenced by the introduction of variants and the use of vaccines.

Finally, information from some countries (such as Brazil, Argentina, Chile, and Colombia) is well-represented in this review. The lack of comprehensive data from other South American countries with poultry ventures (like Peru, Ecuador, Paraguay, Bolivia, and Venezuela) limits a more complete scenario of the IBV molecular epidemiology across the continent. Further studies are needed to obtain information from these countries and assess the effect of IBV on their commercial poultry farms.

## 6. Concluding Remarks

Effective poultry health management relies heavily on continuous monitoring and characterization of circulating pathogenic IBV strains, enabling the implementation of targeted control strategies. IBV identification through molecular surveillance assists in anticipating potential outbreaks and guiding the selection of appropriate vaccine strains.

More recently, advanced techniques such as next-generation sequencing (NGS) have been used to sequence complete IBV genomes from isolates as well as directly from tissue samples and complex matrices [10,24]. Detailed analysis of recombination events has been demonstrated in different lineages, such as GI-1 and GI-23, highlighting how these events shape viral diversity and may impact vaccine control [54]. In addition, recombination between field isolates and vaccine strains has been reported, suggesting the influence of vaccination on virus evolution [55].

These technological advances can be applied to conduct more comprehensive molecular epidemiological surveillance studies and better clarify the evolution and current status of IBV in commercial poultry farms in South America. Furthermore, the introduction of “next generation” technologies in the routine analysis of IBV outbreaks will be essential to implement even timelier biosecurity measures and revise vaccination plans, minimizing economic losses and improving the health and productivity of poultry flocks.

## Data Availability

No new data were created or analyzed in this study. Data sharing is not applicable to this article.
